# Evaluation of Facial Aesthetic Changes in Growing Class II Patients Treated with Herbst or Elastodontics: A Retrospective Study

**DOI:** 10.3390/dj12120411

**Published:** 2024-12-17

**Authors:** Domenico Ciavarella, Rossella Luciano, Mauro Lorusso, Angela Pia Cazzolla, Michele Laurenziello, Carlotta Fanelli, Silvia Caruso, Michele Tepedino

**Affiliations:** 1 Department of Clinical and Experimental Medicine, University of Foggia, 71122 Foggia, Italy; domenico.ciavarella@unifg.it (D.C.); mauro.lorusso@unifg.it (M.L.); angelapia.cazzolla@unifg.it (A.P.C.); michele.laurenziello@unifg.it (M.L.); carlotta.fanelli@unifg.it (C.F.); 2 Department of Biotechnological and Applied Sciences, University of L’Aquila, 67100 L’Aquila, Italy; silvia.caruso@univaq.it; 3 Department of Clinical Medicine, Public Health, Environmental Life Sciences, University of L’Aquila, 67100 L’Aquila, Italy; michele.tepedino@univaq.it

**Keywords:** facial aesthetic, profile changes, Herbst, elastodontics

## Abstract

**Objective:** The objective of this study was to evaluate the facial profile changes of patients treated for class II skeletal malocclusions with an elastodontic appliance compared to those treated with the Herbst appliance and a control group. **Methods:** Forty class II patients were treated using an elastodontic appliance (Group EA) and were compared to 40 patients treated with the Herbst appliance (Group H) and to 40 untreated class II children (Group C). Aesthetic profile variables were analysed using Arnett’s analysis. Cephalograms were compared pre-treatment (T0) and post-treatment (T1). The Wilcoxon signed-rank test or paired-samples *t*-test was used for pairwise comparison of cephalometric measurements taken at T0 and T1. One-way ANOVA and Tukey’s post hoc test were performed to assess differences between the groups. **Results:** In the elastodontic group, the inclination of the upper incisors increased by 4.05°. In addition, the Pog–TVL and B–TVL distances decreased by 2.84 mm and 1.79 mm, respectively. In patients treated with an elastodontic appliance, the inclination of the upper incisors increased by 4.05°. In addition, the Pog–TVL and B–TVL distances decreased by 2.84 mm and 1.79 mm, respectively. In patients treated with the Herbst appliance, the inclination of the lower incisors increased by 6.11°. Furthermore, the treatment resulted in reductions in the Pog–TVL distance (2.58 mm), the B–TVL distance (2.26 mm), and the LL–TVL distance (2.31 mm). **Conclusions:** The profile changes achieved by both devices are favourable for correcting class II skeletal malocclusion.

## 1. Introduction

In orthodontics, it is very common to treat patients with a diagnosis of class II dentoskeletal malocclusion, as it has an incidence of 19.56% in the global population [1] and 12–32% in the Caucasian population [2]. According to Angle [3], class II dental malocclusion is characterised by the distal position of the lower first molar in relation to the upper first molar. Patients with class II division 1 malocclusion have protruding upper incisors, a reduced mentolabial angle, a retruded lower lip, a non-prominent chin, and a convex and retrognathic facial profile [4,5]. The main cause of class II dentoskeletal malocclusion is mandibular retrognathia, which is found in 48% of young patients with class II malocclusion [4]. As is well known from studies by Moss [6], mandibular retrognathia is influenced by the function of the surrounding soft tissues. To correct Class II malocclusion, various therapeutic approaches can be used, including extra-oral traction, a fixed appliance, extraction procedures, and functional orthopaedic appliances [4]. In the literature, different functional orthopaedic appliances have been proposed to induce skeletal and neuromuscular adaptations in order to achieve mandibular growth [7]. Some of these include Herbst, twin-block, elastodontic, and Frankel appliances, as well as Sander’s bite-jumping appliance and the SOCIA (swallowing occlusal contact intercept appliance) [8,9,10,11,12].

The Herbst appliance is a rigid, fixed functional orthodontic appliance widely used for the treatment of Class II malocclusion [13]. It corrects sagittal skeletal and occlusal relationships, stimulates the condylar growth upward and backward, and promotes advancement of the mandibular body, the chin prominence, and the lower lip to improve the aesthetics of the facial profile [8,14,15]. Starting from Pancherz’s original design, this device has been modified, and several variants are now available [16,17]. The main limitation of the original device was related to the headgear effect on the upper molars and the excessive proclination of the lower incisors. Although these limitations have been addressed through the use of TADs [18], the device is still inconvenient and uncomfortable for patients. Additionally, it should be noted that many patients’ parents do not approve of the use of miniscrews, which significantly limits the control of undesirable effects.

Elastodontic devices are removable silicone elastomer devices that are used in patients with permanent early teething to treat Class II malocclusion [19,20]. These devices are suitable for use in children without requiring impressions, as the appropriate size is selected to fit each patient’s mouth. Capable of both dental and orthodontic action, they have recently gained acceptance as a simple, comfortable technique that promotes greater compliance among young patients. Elastodontic devices serve as valuable aids for guiding tooth eruption, being designed to support the ideal positioning of the tongue and perioral muscles [21].

When used during the initial stage of mixed teething, they act as a “shield” for the cheeks and lips; prevent incorrect positioning of the tongue and lower lip during swallowing; produce transverse bone growth; promote nasal breathing; correct open bite, overjet, overbite, and sagittal discrepancy; and improve the relationship between the jaws. [20,22]. Because elastodontic appliances have multiple uses, are easy to use, safe, and well-accepted by patients, they are becoming increasingly popular in clinical practice, not only among orthodontic specialists but also among general dental practitioners. In addition, due to their low cost, elastodontic treatments are also suggested as a means to provide orthodontic care for patients with limited or low economic resources [23].

Individuals with Class II malocclusion require orthodontic treatment for aesthetic improvement because, due to the increased overjet and unfavourable profile, they often have low self-esteem. Facial aesthetics therefore influence the social life of patients [24]. For this reason, changes in facial aesthetics must be evaluated when choosing one therapeutic approach over another. Calculating these changes requires the use of the proportional relationships of the soft tissues, as well as cephalometric references that relate teeth position to cranial or facial bones [15]. Although there are several studies that examine the dental, skeletal, and aesthetic effects of each appliance separately [5,8,11], there have been no studies that have analysed these effects simultaneously in patients treated with the Herbst appliance compared to patients treated with elastodontics.

The aim of this study was to evaluate modifications in facial profile aesthetics following Herbst and elastodontic therapy in patients with Class II malocclusion compared to a control group. The null hypothesis tested was that the profile changes obtained with Herbst and elastodontics therapy were not significantly different compared to those in the untreated control group.

## 2. Materials and Methods

This study was reported following the Strengthening the Reporting of Observational Studies in Epidemiology (STROBE) guidelines for observational studies [25].

All procedures in this research protocol adhere to the Declaration of Helsinki and have been approved by the Ethics Committee of the University of Foggia (Approval no.43/CE/2019). The records were retrieved retrospectively and analysed anonymously, and the patients signed a written informed consent form. The inclusion criteria were class II skeletal malocclusion (ANB > 4°), mandibular retrognathia (SNB < 78°), age between 8 and 11 years, overjet ≥ 5 mm, late mixed dentition, absence of temporomandibular joint disorders, and skeletal age between CS2 and CS3 according to the cervical vertebral maturation method. The exclusion criteria were destructive caries, skeletal malformation, mono or bilateral cross bite and scissor bite, systematic diseases, a congenitally missing or extracted permanent tooth, previous orthodontic treatment, and impacted teeth.

A power analysis (G*Power 3.1.9.2, Franz Faul, Universität Kiel, Germany) showed that to detect a large effect size of 0.4 [26] with a one-way ANOVA test with an α error probability of 0.05 and a power of 0.95, 34 subjects for each group would be needed.

The sample consisted of three groups: one group composed of patients treated with the Herbst appliance (Group H), one group composed of patients treated with an elastodontic appliance (Group EA), and one group composed of untreated controls (Group C). Groups H and EA were retrospectively enrolled from patients treated at the Department of Orthodontics, University of Foggia, Italy, in chronological order from October 2018 to July 2020. Once a Class I molar and canine relationship was attained, the treatment reached its conclusion. Group C was selected from the Michigan Medical Library. Similar dentoskeletal characteristics were observed in the three samples at baseline (T0). The records included pre-treatment (T0) and post-treatment (T1) assessments comprising study models, photographs, panoramic radiographs, and lateral cephalograms.

### 2.1. Group H

**Figure 1:**
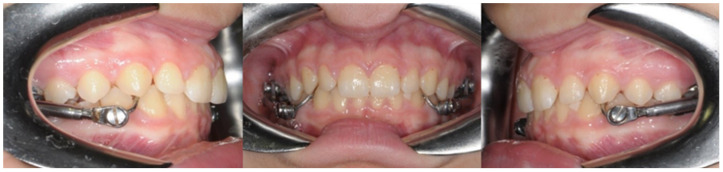
The clinical photos of the Herbst appliance.

The group treated with the Herbst appliance (Group H) consisted of 16 females and 24 males with a mean age of 9.2 ± 0.6 years. Figure 1 shows the clinical photos of the Herbst appliance. The mean treatment time was 10 months. The Herbst appliance was composed of a telescopic tube attached to the band of the superior first permanent molar and by a telescope plunger attached to the inferior canine. In the maxillary arch, the anchorage was provided by a palatal or buccal sectional arch wire connecting the first molar to the first premolar. Additionally, a stainless-steel lingual arch was used to connect the left mandibular molars to the right mandibular molars, with occlusal rests on the lower first premolars or deciduous second molars.

### 2.2. Group EA

**Figure 2:**
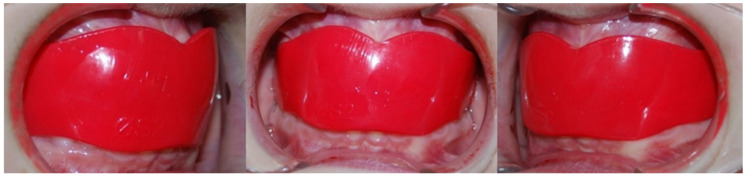
The clinical photos of the Elastodontic appliance.

The group treated with the elastodontic appliance (Group EA) consisted of 22 females and 18 males with a mean age of 9.46 ± 0.6 years. The mean treatment time was 14 months. The EAs used (AMCOP SC series, Micerium, Genoa, Italy) were preformed elastomeric silicone appliances. To select the size, patient impressions were evaluated, and the distance between the vestibular cusps of the upper first molars was measured. This measurement was then compared to a size selection chart provided by the manufacturer. Patients were instructed to wear the appliance during sleep and for four hours in the afternoon. The patients were not asked to perform myofunctional exercises. Figure 2 shows the clinical photos of the elastodontic appliance. Patients treated with elastodontic devices were monitored every two weeks to ensure proper use of the device for 12 h a day.

### 2.3. Group C

The control group (Group C) consisted of 26 females and 14 males with a mean age of 9.7 ± 0.6 years. These subjects did not receive any orthodontic treatment. These patients were sourced from the Michigan Medical Library and specifically chosen based on age and gender to ensure comparability with the other two analysed groups.

### 2.4. Cephalometric Analysis

Lateral head films (Gendex Dental System, Varese, Italy, Gendex GXDP-700) were taken with the patient’s head correctly positioned in a cephalostat, ensuring centric occlusion and providing ample visibility of reference structures without significant head rotation. To minimise methodological errors, cephalometric analysis was performed by a qualified examiner, and all measurements were conducted twice by the same operator.

**Figure 3:**
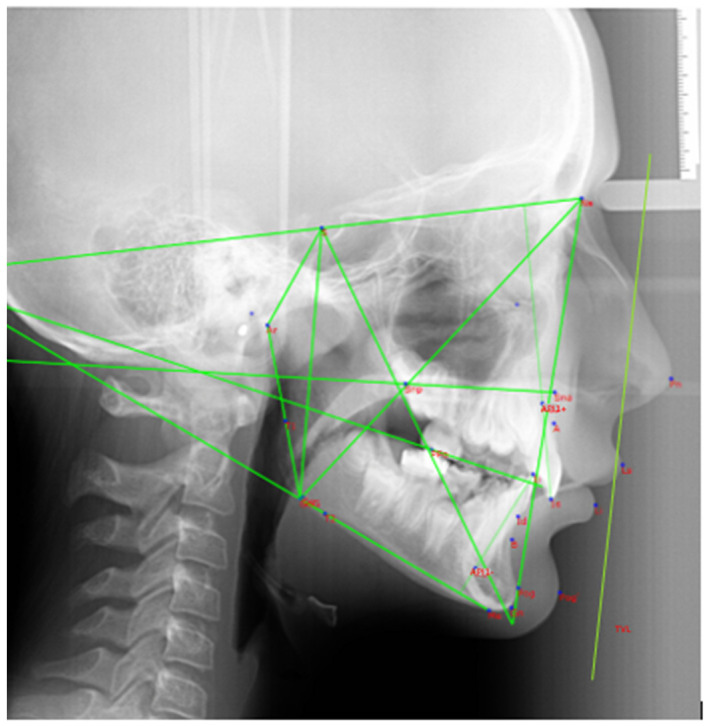
Cephalometric landmarks and reference lines.

Facial profile assessments were carried out using the Arnett analysis [27]. This analysis is based on linear measurements and angles, with reference to a line called the true vertical line (TVL). The TVL is a vertical line perpendicular to the Frankfurt plane, which passes through the subnasal point (SN). The landmarks and reference lines used in the cephalometric analysis are presented in Figure 3 and described in Table 1.

### 2.5. Statistical Analysis

Data distribution analysis was conducted using the Shapiro–Wilk normality test (Table 2). Descriptive statistics were also performed (Table 2). The Wilcoxon signed-rank test (Table 2) was used for pairwise comparison of the cephalometric measurements taken at T0 (pre-treatment) and at T1 (post-treatment) within each group, while a paired sample *t*-test was used in the case of a normal distribution of the data. The data were analysed using the GraphPad Prism software, version 6.0 (GraphPad Prism Software, San Diego, CA, USA). A *p*-value of <0.05 was considered to be statistically significant. The differences between the three groups were evaluated by a one-way ANOVA test for the T1 − T0 difference of each variable, followed by Tukey’s post hoc test (Table 3 and Table 4).

At baseline (T0), the Herbst group showed more proclined upper and lower incisors compared to the elastodontic group. Consequently, the UL–TVL and 1+TVL distances were lower in the Herbst group. In addition, the B–TVL, POG–TVL, and LL–TVL distances were smaller in the Herbst group than in the elastodontic group.

## 3. Results

### 3.1. Dental Outcomes

The EA group showed an increase in the 1+SN angle (*p* < 0.01). The H group showed an increase in the IMPA (*p* < 0.01) and a decrease in the 1+SN angle (*p* < 0.01).

### 3.2. Aesthetic Outcomes

The C group (Table 2) showed a decrease in the linear distance of the LL–TVL (*p* < 0.01) and a decrease in the POG’–TVL distance (*p* < 0.05). The EA Group showed a significant reduction in the B’–TVL (*p* < 0.01) and POG’–TVL (*p* < 0.01) distances. The H group had a reduction in the LL–TVL (*p* < 0.01), B’–TVL (*p* < 0.05), and POG’–TVL (*p* < 0.05) distances.

**Table 2:** Descriptive statistics, Wilcoxon signed-rank test and *t*-test between cephalometric variables taken at T0 and at T1 within the three groups.

	Group C		GROUP EA		GROUP H	
	T0	T1	T0	T1	T0	T1
**1+SN**						
Median	103.72	106.12	97.96	101.10	109.50	108.20
Mean	105.90	107	96.85	100.90	110.00	107.50
Std. deviation	4.82	5.80	10.84	7.77	7.74	6.42
Std. error of mean	1.07	1.29	2.42	1.73	1.73	1.43
Normality test	N.S.	N.S.	<0.05	<0.05	N.S.	<0.05
*p* value	N.S.		*p* < 0.01		*p* < 0.01	
**IMPA**						
Median	92.13	93.25	95.49	96.58	95.47	100.70
Mean	90.85	91.18	95.98	97.20	94.79	100.90
Std. deviatiom	6.48	7.24	3.09	6.34	3.99	3.45
Std. error of mean	1.45	1.62	0.69	1.41	0.89	0.77
Normality test	<0.05	<0.05	<0.05	<0.05	N.S.	N.S.
*p* value	N.S.		N.S.		*p* < 0.01	
**1+TVL**						
Median	−10.65	−11.89	−9.89	−9.41	−8.08	−9.39
Mean	−11.98	−10.57	−9.66	−8.83	−8.33	−9.11
Std. deviation	2.82	2.54	2.46	2.24	2.02	1.50
Std. error of mean	0.63	0.57	0.55	0.50	0.45	0.33
Normality test	<0.05	<0.05	N.S.	N.S.	N.S.	N.S.
*p* value	N.S.		N.S.		N.S.	
**SupportLab–TVL**						
Median	−0.21	0.02	−0.03	−0.08	−0.66	−0.28
Mean	−0.24	0.04	0.36	0.04	−0.44	−0.08
Std. deviation	0.68	2.0	1.74	0.93	0.86	1.01
Std. error of mean	0.15	0.44	0.39	0.20	0.19	0.22
Normality test	<0.05	<0.05	N.S.	N.S.	N.S.	<0.05
*p* value	N.S.		N.S.		N.S.	
**UL–TVL**						
Median	1.29	0.68	2.33	1.76	0.80	0.88
Mean	1.49	0.71	2.09	2.04	1.05	1.37
Std. deviation	1.92	3.26	2.13	2.26	1.13	1.31
Std. error of mean	0.42	0.72	0.47	0.50	0.25	0.29
Normality test	N.S.	N.S.	<0.05	N.S.	N.S.	N.S.
*p* value	N.S.		N.S.		N.S.	
**LL–TVL**						
Median	−5.17	−3.43	−2.86	−1.19	−2.71	0.18
Mean	−5.44	−3.77	−2.33	−1.42	−2.82	−0.51
Std. deviation	400	3.48	2.96	3.35	1.89	2.21
Std. error of mean	0.89	0.77	0.66	0.75	0.42	0.49
Normality test	<0.05	<0.05	<0.05	N.S.	N.S.	N.S.
*p* value	*p* < 0.01		N.S.		*p* < 0.01	
**B’–TVL**						
Median	−15.26	−13.31	−11.65	−9.58	−12.27	−8.83
Mean	−15.81	−13.86	−12.19	−10.40	−12.33	−10.07
Std. deviation	5.12	3.74	2.96	3.95	2.55	350
Std. error of mean	1.14	0.83	0.66	0.88	0.57	0.78
Normality test	<0.05	<0.05	N.S.	N.S.	N.S.	N.S.
*p* value	N.S.		*p* < 0.01		*p* < 0.05	
**POG’–TVL**						
Median	−18.93	−15.32	−13.63	−10.19	−11.95	−7.57
Mean	−17.73	−14.43	−12.85	−10.01	−12.86	−10.28
Std. deviation	5.42	5.49	342	3.51	4.30	5.98
Std. error of mean	1.21	1.22	0.76	0.78	0.96	1.33
Normality test	N.S.	N.S.	<0.05	<0.05	N.S.	N.S.
*p* value	*p* < 0.05		*p* < 0.01		*p* < 0.05	

N.S.: non significant.

### 3.3. Comparison of Outcomes Between Groups

One-way ANOVA (Table 3) showed a statistically significant difference in the following variables: 1+SN, IMPA,1+TVL, UL–TVL, and LL–TVL. Tukey’s post hoc test (Table 4) showed significant differences as follows:

− 1+SN: 3.85° greater in Group C than in Group H and 4.47° greater in Group EA than in Group H.
− IMPA: 5.73° greater in Group H than in Group C and 4.48° greater in Group H than in Group EA.
− 1+TVL: 2.19° greater in Group H than in Group C.
− UL–TVL: 2.29° greater in Group C than in Group H and 1.71° greater in Group EA than in Group H.
− LL–TVL: 2.72° greater in Group H than in Group EA.

**Table 3:** One-way ANOVA test for all the cephalometric variables between the three groups.

		Sum of Squares	df	Mean Square	F	Sig.
1+SN	Between groups	234.82	4	117.41	7.294	0.002 *
1-GOME(IMPA)	Between groups	381.28	4	190.61	8.49	0.001 *
1+TVL	Between groups	51.52	4	25.76	4.32	0.018 *
SUPPORTLAB–TVL	Between groups	5.45	4	2.72	1.06	0.353
UL–TVL	Between groups	56.81	4	28.40	14.57	0.000 *
LL–TVL	Between groups	74.26	4	37.13	5.60	0.006 *
B’–TVL	Between groups	5.38	4	2.69	0.21	0.805
POG’–TVL	Between groups	10.90	4	5.45	0.29	0.750

*: *p* < 0.05.

**Table 4:** Tukey’s post hoc test.

Dependent Variable	(I) Group	(J) Group	Mean Difference (I − J)	Std. Error	Sig.	95% Confidence Interval	
						Lower Bound	Upper Bound
1+SN	C	H	3.85 *	1.26	0.01	0.79	6.90
	EA	C	0.62	1.26	0.87	−2.42	3.67
	EA	H	4.47 *	1.26	0.00	1.42	7.25
1-GOME (IMPA)	C	H	−5.73 *	1.49	0.00	−9.34	−2.13
	EA	C	0.89	1.49	0.82	−2.70	4.50
	EA	H	−4.84 *	1.49	0.00	−8.44	−1.23
1+TVL	C	H	−2.19 *	0.77	0.01	0.33	4.05
	EA	C	−0.59	0.77	0.72	−2.45	1.26
	EA	H	1.60	0.77	0.10	−0.25	3.45
SUPPORTLAB–TVL	C	H	−0.10	0.50	0.97	−1.32	1.11
	EA	C	−0.57	0.50	0.49	−1.79	0.64
	EA	H	−0.68	0.50	0.37	−1.90	0.53
UL–TVL	C	H	2.29 *	0.44	0.00	1.22	3.35
	EA	C	−0.57	0.44	0.39	−1.63	0.48
	EA	H	1.71 *	0.44	0.00	0.65	2.77
LL–TVL	C	H	−1.33	0.81	0.23	−3.29	0.62
	EA	C	−1.38	0.81	0.21	−3.34	0.56
	EA	H	−2.72 *	0.81	0.00	−4.68	−0.76
B’–TVL	C	H	−0.30	1.11	0.96	−2.97	2.37
	EA	C	−0.42	1.11	0.92	−3.10	2.24
	EA	H	−0.73	1.11	0.79	−3.40	1.94
POG’–TVL	C	H	1.04	1.37	0.72	−2.25	4.34
	EA	C	−0.49	1.37	0.93	−3.79	2.80
	EA	H	0.54	1.37	0.91	−2.75	3.85

*: *p* < 0.05.

According to the results of the study, the null hypothesis was rejected.

## 4. Discussion

The purpose of orthodontic treatment is to achieve a good occlusal relationship with facial harmony, which are both determined by the hard and soft tissues of the face [28]. The treatment plan for class II malocclusion should, therefore, aim to resolve dental and skeletal disharmony in order to achieve a favourable facial aesthetic [29]. It is not possible to define the characteristics of an attractive soft tissue profile, but according to some authors [15], a relatively straight profile is preferred. Since Class II malocclusion is characterised by a convex facial profile of the soft tissues, orthodontic treatment should enhance facial aesthetics by reducing the convexity of the profile [15].

To address dental discrepancies, clear aligners are increasingly being used. These transparent devices are widely utilised because they meet patients’ aesthetic needs. Furthermore, several studies have demonstrated their effectiveness for correcting Class II dental malocclusion [30,31].

To the best of our knowledge, this is the first study to evaluate changes in facial aesthetics in Class II patients treated with the Herbst appliance, treated with elastodontics, and a control group.

From T0 to T1, elastodontic treatment resulted in a significant increase in 1+SN due to increased labial inclination of the upper incisors, in line with the previous observations by Keski-Nisula et al. [22]. A reduced 1+TVL distance is also indicative of labial inclination of the upper incisors, which can worsen the patients’ facial profile. However, Galluccio et al. [32] reported significant retraction of the upper lip relative to Ricketts’ aesthetic line in Class II subjects treated with the Occlus-O-Guide.

However, from T0 to T1, elastodontic treatment was also associated with an increase in IMPA due to the increase in labial inclination of the lower incisors, which also resulted in the advancement of the lower lip as indicated by a reduction in the LL–TVL distance, which, nevertheless, was not statistically significant. Furthermore, a reduction in the B’–TVL and POG’–TVL distances was observed in patients treated with elastodontic appliances. Therefore, elastodontic treatment resulted in a change in the facial profile of the patients, leading to an improvement of the lower third of the face.

Patients treated with the Herbst appliance experienced greater discomfort, including irritation, pain, and difficulty speaking and eating. However, the device provided more precise and constant control over tooth movements and required less active effort from the patients, although they showed more difficulty maintaining appropriate oral hygiene. The elastodontic appliance required good cooperation from the patients, who had to wear it for the number of hours per day indicated by the orthodontist. However, patients had no difficulty managing oral hygiene and experienced less discomfort and pain during therapy.

In patients treated with Herbst’s appliance, B’–TVL, POG’–TVL, and LL–TVL decreased significantly from T0 to T1, while an increase in IMPA related to the labial inclination of the lower incisors was observed. These patients also showed a decrease in 1+SN due to the palatal inclination of the upper incisors. These results are in agreement with those reported by Pancherz et al. [15] in a group of patients with Class II malocclusion treated with the Herbst appliance, where the E-line was used to assess soft tissue-related changes. Therefore, the Herbst appliance results in an advancement of the mandible, a proclination of the lower incisors, and a palatal inclination of the upper incisors, which result in a reduction in the convexity of the profile by improving the skeletal and soft tissue relationships of the middle third of the face, as reported by Irezli and Baysal [33].

Different studies have reported an increase in mandibular length after Herbst treatment [34,35]. However, several limitations have been observed with the use of a Herbst appliance. One of the primary issues is the headgear effect, which results in upper molar distalization and intrusion, leading to increased facial divergence due to mandibular molar extrusion [36,37]. This results in an increase in lower facial height and can lead to a worsening of divergence. Therefore, special care should be taken, especially with hyperdivergent patients with an open bite [38].

When comparing the groups, a greater inclination of the upper incisors was observed in patients treated with elastodontics compared to those treated with the Herbst appliance, while a greater labial inclination of the lower incisors was observed in patients treated with the Herbst appliance compared to those treated with elastodontics. The effects observed on soft tissues reflect the action of the appliances on the teeth and hard tissue; in fact, the UL–TVL distance was increased in patients treated with elastodontics compared to those treated with the Herbst appliance. Finally, the LL–TVL distance was greater in patients treated with the Herbst appliance compared to those treated with elastodontics.

Elastodontic appliances can be used for different purposes. However, it is important to note that these appliances can improve respiratory alterations. Patano et al. [39] observed that elastodontic therapy can result in significant airway changes and an inferior positioning of the hyoid bone in skeletal Class II malocclusion patients, along with improvements in phonation and deglutition. For this reason, it is important to consider the favourable effects of elastodontic appliances, which can enhance patients’ quality of life.

The results of the present study show that both devices resulted in an improvement in soft tissue by reducing the profile convexity in patients with Class II malocclusion. It is likely that the effect on soft tissue is, in part, due to the action of the devices on the muscles, as a change in neuro-muscular circuits promotes a balancing of hard tissue and consequently improves soft tissue projection.

### Limitations of the Study

The limitations of the study are related to its retrospective nature and lack of long-term follow-up. Moreover, when comparing a fixed and a mobile device, the results observed in the group of patients treated with elastodontic devices could be influenced by patient compliance. As the untreated subjects were recruited from a university dental clinic, some inherent biases are possible. It is important to point out that functional devices primarily have dentoalveolar effects. For this reason, their impact on soft tissue might be limited, especially in the long term.

Finally, initiating Herbst therapy during mixed dentition may have limited the skeletal effects of the device. However, the risk associated with trauma related to the proclination of the upper incisors and increased overjet, combined with the associated psychological disorders, made it necessary to start therapy early.

Long-term clinical studies with a large sample are therefore required to overcome these limitations.

## 5. Conclusions

Elastodontics are removable devices whose effectiveness is related to patient compliance, as opposed to the Herbst device, which is cemented and therefore fixed. In the present study, patients treated with elastodontic appliances showed greater upper incisor inclination, while greater labial inclination of the lower incisors was observed in patients treated with the Herbst appliance. Specifically, the UL–TVL distance increased in patients treated with elastodontic appliances, whereas the LL–TVL distance was greater in patients treated with the Herbst appliance. Both devices are effective in correcting class II malocclusions. These devices are able to modify soft tissues and thus improve patient profiles. However, a comparison of these devices reveals that dentoalveolar effects are associated with soft tissue changes. Therefore, it is essential to make an accurate diagnosis to plan appropriate therapeutic treatment.

## Figures and Tables

**Table 1:** Cephalometric measurements: dental and aesthetic parameters.

**Dental Measurements**	
1+SN	Basal incisor angle between the S–N line and the straight line passing between the incisal edge and API+ (apical point upper incisor)
IMPA	Incisor angle between the line passing through the lower incisor margin and API- (apical point lower incisor) and the Go–Me line
1+TVL	Linear distance between the most vestibular point of 1+ and the TVL
**Aesthetic Measurements**	
SupportLab–TVL	Linear value of the distance between the lip support point and the true vertical line
UL–TVL	Linear value of the distance between the most protruding point of the upper lip and the true bertical line
LL–TVL	Linear value of the distance between the most protruding point of the lower lip and the true vertical line
B’–TVL	Linear value of the distance between the most recessed point of the chin and the true vertical line
POG’–TVL	Linear value of the distance between the most protruding point of the chin and the true vertical line

## Data Availability

The raw data supporting the conclusions of this article will be made available by the authors on request.
